# Hyperthermic Intravesical Chemotherapy as a Bladder-Sparing Alternative to BCG in the Management of Non-Muscle-Invasive Bladder Cancer: A Systematic Review of Oncological Outcomes in the Era of BCG Shortage

**DOI:** 10.3390/jcm15166352

**Published:** 2026-08-17

**Authors:** Alexei Croitor, Victor Cadariu-Brailoiu, Andrei-Dan Zbircea, Razvan Bardan, Vlad Dema, Sorin Dema, Alis Dema, Alin Cumpanas

**Affiliations:** 1 Department XV, Discipline of Urology, Faculty of Medicine, “Victor Babes” University of Medicine and Pharmacy, 300041 Timisoara, Romania; alexei.croitor@umft.ro (A.C.); victor.cadariu-brailoiu@rezident.umft.ro (V.C.-B.); razvan.bardan@umft.ro (R.B.); cumpanas.alin@umft.ro (A.C.); 2 Oncology and Radiotherapy Department, Municipal Emergency Clinical Hospital, 300254 Timisoara, Romania; sorin.dema@umft.ro; 3 Department of Microscopic Morphology—Pathology, Faculty of Medicine, “Victor Babes” University of Medicine and Pharmacy, 300041 Timisoara, Romania; dema.alis@umft.ro; 4 Department of Pathology, “Pius Brinzeu” Clinical Emergency Hospital, 300723 Timisoara, Romania

**Keywords:** non-muscle-invasive bladder cancer, hyperthermic intravesical chemotherapy, chemohyperthermia, mitomycin C, Bacillus Calmette–Guérin, recurrence-free survival, bladder preservation, BCG shortage

## Abstract

**Background/Objectives**: The ongoing Bacillus Calmette–Guérin (BCG) shortage, treatment intolerance, and BCG-unresponsive disease necessitate bladder-sparing alternatives for non-muscle-invasive bladder cancer (NMIBC). We reviewed the clinical evidence for hyperthermic intravesical chemotherapy (HIVEC). **Methods**: PubMed and PubMed Central were searched from inception to 15 March 2026 (updated 10 July 2026) for primary studies of device-assisted HIVEC in NMIBC. Studies reporting recurrence-free survival (RFS), progression, response, or safety were eligible. Records were screened and data extracted independently in duplicate, and risk of bias was assessed with RoB 2 (randomised trials), ROBINS-I (non-randomised comparative studies), and the Newcastle–Ottawa Scale (single-arm and registry cohorts). Outcomes were synthesised narratively within pre-specified clinical strata, with carcinoma in situ (CIS) analysed separately from papillary-only disease. **Results**: Twenty-four studies (>3600 patients), including seven randomised trials and a large multinational registry, were included. In BCG-naive or mixed-risk populations, 12-month RFS generally ranged from 78% to 98% and 24-month RFS from 57% to 88%; progression was uncommon. In BCG-unresponsive or BCG-failure disease, 24-month RFS was typically 40–60%, with poorer control in carcinoma in situ. Comparative studies mostly reported no statistically significant difference in RFS or progression versus BCG, but these analyses were predominantly retrospective or underpowered, and the single randomised signal favouring HIVEC over BCG was confined to a per-protocol analysis. Two randomised trials against non-heated mitomycin C (HIVEC-1 and HIVEC-II) found no recurrence benefit from hyperthermia, whereas an earlier randomised trial of radiofrequency thermochemotherapy reported a large long-term advantage over mitomycin C alone. Severe adverse events were infrequent, including approximately 2% in the largest series. **Conclusions**: HIVEC appears to be a tolerable bladder-sparing option in selected intermediate- and high-risk NMIBC. The available evidence is compatible with, but does not establish, equivalence or non-inferiority to BCG, because most comparative data are non-randomised, underpowered, and at moderate-to-serious risk of bias. Disease control after BCG failure is clinically meaningful but less durable, particularly in CIS. Adequately powered randomised trials reporting intention-to-treat outcomes are needed.

## 1. Introduction

Bladder cancer is among the most frequently diagnosed malignancies worldwide and imposes a substantial and growing burden on health systems, with incidence and mortality continuing to rise in absolute terms as populations age [1]. The majority of patients present with disease confined to the urothelium or lamina propria, collectively termed non-muscle-invasive bladder cancer (NMIBC), which encompasses stage Ta, T1, and carcinoma in situ (CIS) and represents approximately three-quarters of incident bladder cancer at diagnosis [2]. Although the immediate prognosis for survival is favourable relative to muscle-invasive disease, the natural history of NMIBC is dominated by a high propensity for recurrence and, in higher-risk categories, progression to muscle-invasive disease [2]. This biological behaviour commits patients to repeated cystoscopic surveillance, multiple resections, and prolonged intravesical therapy, making bladder cancer one of the most resource-intensive malignancies to manage over a lifetime [1,2].

The dominant modifiable risk factor for bladder cancer is cigarette smoking, which accounts for roughly half of cases and approximately triples the risk relative to never smoking, underscoring the importance of primary prevention alongside therapeutic innovation [3]. Diagnosis rests on cystoscopy and histological evaluation of tissue obtained by transurethral resection of the bladder tumour (TURBT), with enhanced visualisation technologies such as photodynamic (blue-light) cystoscopy and narrow-band imaging improving lesion detection and reducing early recurrence compared with white-light cystoscopy alone [4]. The molecular landscape of NMIBC is increasingly well characterised, with frequent TERT promoter, FGFR3, and chromatin-modifying gene alterations that may eventually inform individualised therapy and response prediction [5]. Accurate staging and grading at first resection are decisive, because residual or under-staged disease drives subsequent recurrence and progression [6].

Because resection alone is insufficient for intermediate- and high-risk disease, adjuvant intravesical therapy is recommended to consolidate the resection and reduce recurrence [6]. Risk-adapted treatment selection is guided by validated prognostic frameworks; the European Organisation for Research and Treatment of Cancer (EORTC) risk tables, derived from 2596 patients, allow individualised estimation of recurrence and progression probabilities based on tumour number, size, prior recurrence rate, stage, grade, and CIS [7]. These tools underpin the contemporary stratification of patients into low-, intermediate-, high-, and very-high-risk categories, which determines whether intravesical chemotherapy, BCG, or early radical cystectomy is advised [6]. The fundamental challenge motivating ongoing research is that even optimally delivered adjuvant therapy leaves a substantial fraction of patients who recur, become unresponsive, or cannot tolerate the available agents [6].

For high-risk disease, intravesical BCG immunotherapy with maintenance has historically delivered the most durable reductions in recurrence and progression, and it remains the guideline-endorsed standard of care [6]. The antitumour effect of BCG depends on a complex, incompletely understood interaction between mycobacterial components and the urothelial immune microenvironment, and its delivery is accompanied by a recognised burden of local and systemic adverse events that can necessitate dose reduction or discontinuation [8]. Despite its efficacy, BCG is encumbered by three increasingly consequential limitations: a protracted global manufacturing shortage that prevents guideline-concordant scheduling, a meaningful rate of intolerance, and a clinically defined subset of patients who develop BCG-unresponsive disease [8,9]. Standardised definitions of BCG-unresponsive, BCG-refractory, BCG-relapsing, and BCG-intolerant disease, articulated by the International Bladder Cancer Group, are now essential for comparing trials and treatments in this space [9].

For BCG-unresponsive disease, radical cystectomy remains the guideline standard, yet it is a morbid operation that many patients are unfit for or decline because of its substantial quality-of-life consequences, creating a strong impetus for bladder-sparing salvage strategies [2]. Several novel agents have emerged for this setting and frame the comparative landscape into which device-assisted therapies must be positioned: the immune checkpoint inhibitor pembrolizumab achieved a 41% complete response rate in BCG-unresponsive CIS [10], intravesical nadofaragene firadenovec gene therapy produced complete responses in just over half of CIS patients [11], and sequential intravesical gemcitabine–docetaxel has been associated with favourable progression outcomes in real-world BCG-unresponsive cohorts [12]. Real-world quality-indicator registries confirm that, in routine practice, a sizeable proportion of high-risk patients experience BCG intolerance or recurrence and require such alternatives [13].

Hyperthermic intravesical chemotherapy (HIVEC), used interchangeably with the terms chemohyperthermia and intravesical thermochemotherapy, is among the most actively investigated device-assisted strategies to address this gap [14]. The approach couples controlled heating of the bladder wall, typically to around 42–43 °C, with intravesical instillation of a cytotoxic agent—most often mitomycin C, but also epirubicin, pirarubicin, or gemcitabine—delivered through external radiofrequency, intravesical radiofrequency, or conductive recirculation platforms [14]. These platforms differ appreciably in heating mechanism, thermometry, consumables, capital cost, and the volume of supporting evidence. The biological rationale is well supported by in vitro work demonstrating synergistic cytotoxicity between mild hyperthermia and several chemotherapeutic agents, mediated by enhanced drug uptake, impaired DNA repair, and protein denaturation [15]. Given the urgency created by the BCG shortage and the proliferation of recent clinical reports, this systematic review consolidates the primary, full-text clinical evidence on HIVEC oncological outcomes in NMIBC into a coherent, stratified evidence map [14].

## 2. Materials and Methods

### 2.1. Protocol, Reporting Framework, and Review Question

**Figure 1:**
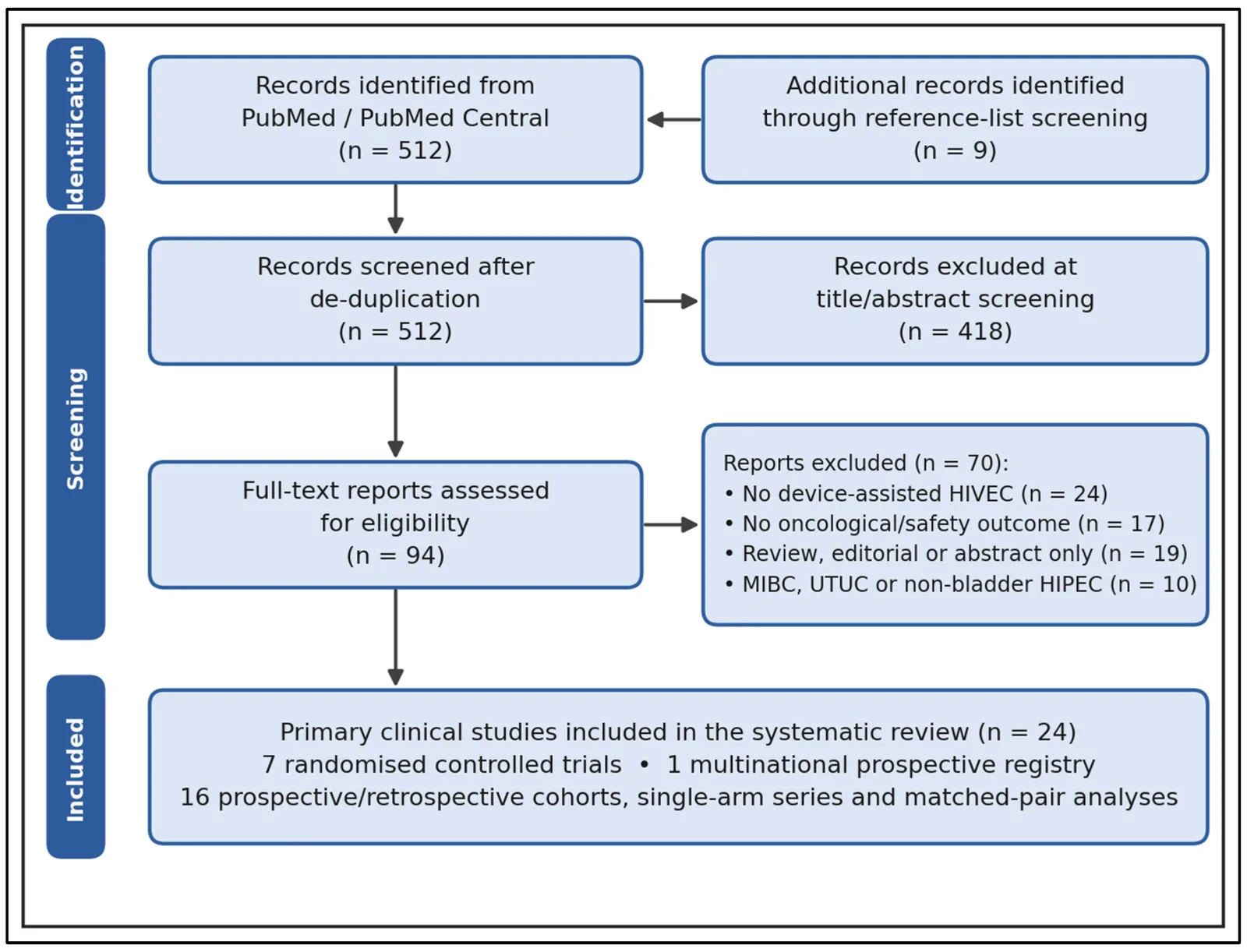
PRISMA 2020 flow diagram of study identification, screening, eligibility assessment, and inclusion.

This systematic review was designed and reported in accordance with the principles of the Preferred Reporting Items for Systematic Reviews and Meta-Analyses (PRISMA) 2020 statement [16], adapted to a narrative-synthesis review of primary clinical studies, and the study-selection process is summarised in a PRISMA flow diagram (Figure 1). The completed PRISMA 2020 checklist is provided in the Supplementary Materials (Table S1). The review was not prospectively registered in PROSPERO, and no separate protocol was published; the eligibility criteria, outcomes, and synthesis plan described below were nevertheless fixed before data extraction began, and this absence of prospective registration is acknowledged in Section 5. The review question was formulated using a PICO (Population, Intervention, Comparator, and Outcome) structure. The population of interest comprised adult patients with histologically confirmed non-muscle-invasive bladder cancer (stages Ta, T1, and/or carcinoma in situ) across all risk strata, including BCG-naive patients, patients with BCG intolerance, and patients with BCG-unresponsive or BCG-failure disease. The intervention of interest was device-assisted hyperthermic intravesical chemotherapy (HIVEC), encompassing the synonymous concepts of chemohyperthermia, intravesical thermochemotherapy, and radiofrequency-induced thermochemotherapy, delivered with any cytotoxic agent (mitomycin C, epirubicin, pirarubicin, or gemcitabine) and any clinically validated heating platform. The comparator, where present, was intravesical BCG, conventional (non-heated) intravesical chemotherapy, or an alternative HIVEC schedule; single-arm studies without a comparator were also eligible. The primary outcomes were recurrence-free survival (RFS) and progression-free survival (PFS); secondary outcomes included complete response rate, cancer-specific and overall survival, bladder-preservation rate, and treatment-related adverse events. The overarching objective was to characterise the effectiveness and tolerability of HIVEC as a bladder-sparing strategy, with particular attention to its role in the context of the global BCG shortage. The review was conceived as a focused synthesis of primary evidence rather than a quantitative meta-analysis, given anticipated clinical and methodological heterogeneity among eligible studies.

### 2.2. Information Sources and Search Strategy

A systematic search of the PubMed database, including its PubMed Central full-text repository (National Library of Medicine, Bethesda, MD, USA; https://pubmed.ncbi.nlm.nih.gov/, accessed on 10 July 2026), was conducted to identify all relevant primary clinical studies. The search combined controlled vocabulary and free-text terms describing the intervention and the disease. Intervention terms included “hyperthermic intravesical chemotherapy,” “HIVEC,” “chemohyperthermia,” “thermochemotherapy,” “radiofrequency-induced thermochemotherapy,” and “device-assisted intravesical therapy,” each combined with the chemotherapeutic agents “mitomycin,” “epirubicin,” “pirarubicin,” and “gemcitabine.” Disease terms included “non-muscle-invasive bladder cancer,” “NMIBC,” “bladder cancer,” “urothelial carcinoma,” and “carcinoma in situ.” Outcome terms such as “recurrence-free survival,” “progression,” “recurrence,” and “Bacillus Calmette–Guérin” were used to refine retrieval. Boolean operators (AND, OR) were applied to combine concept blocks, and the database’s automatic term mapping was leveraged to capture synonyms and lexical variants. Several complementary queries were executed to maximise sensitivity, including searches anchored on “HIVEC,” on “chemohyperthermia mitomycin recurrence-free survival,” and on BCG-status qualifiers such as “BCG-naive,” “BCG-unresponsive,” and “BCG shortage.” Reference lists of retrieved studies and relevant reviews were screened to identify additional eligible reports. No lower date limit and no language restriction were applied. The final electronic search was executed on 15 March 2026 and was re-run on 10 July 2026 during revision to capture newly indexed reports; both runs used the same strategy. The complete PubMed strategy, reproduced verbatim, was: ((“hyperthermic intravesical chemotherapy”[tiab] OR “HIVEC”[tiab] OR “chemohyperthermia”[tiab] OR “chemo-hyperthermia”[tiab] OR “thermochemotherapy”[tiab] OR “thermo-chemotherapy”[tiab] OR “radiofrequency-induced thermochemotherapy”[tiab] OR “bladder hyperthermia”[tiab] OR “device-assisted”[tiab] OR “Hyperthermia, Induced”[Mesh]) AND (“non-muscle-invasive bladder cancer”[tiab] OR “NMIBC”[tiab] OR “bladder cancer”[tiab] OR “bladder neoplasm*”[tiab] OR “urothelial carcinoma”[tiab] OR “carcinoma in situ”[tiab] OR “Urinary Bladder Neoplasms”[Mesh]) AND (“Administration, Intravesical”[Mesh] OR “intravesical”[tiab] OR “mitomycin”[tiab] OR “epirubicin”[tiab] OR “pirarubicin”[tiab] OR “gemcitabine”[tiab] OR “BCG”[tiab])) NOT (“review”[pt] OR “meta-analysis”[pt] OR “editorial”[pt] OR “comment”[pt] OR “case reports”[pt]). This string retrieved 512 records; a further 9 records were identified from the reference lists of retrieved primary studies and relevant narrative reviews. All records were managed and de-duplicated before screening, and bibliographic data for all retrieved records were obtained from PubMed.

### 2.3. Eligibility Criteria and Study Selection

Studies were eligible for inclusion if they met all of the following criteria: (i) they enrolled patients with non-muscle-invasive bladder cancer; (ii) they evaluated device-assisted hyperthermic intravesical chemotherapy as a therapeutic intervention; (iii) they reported at least one clinical oncological outcome, such as recurrence-free survival, progression-free survival, complete response, cancer-specific or overall survival, or a quantified safety endpoint; and (iv) they were reported as a complete primary study rather than as an abstract only. Full-text accessibility was removed as an eligibility criterion during revision; reports that were not freely available were obtained through institutional subscriptions or by author request, and no study was excluded because its full text could not be retrieved. Eligible study designs comprised randomised controlled trials, prospective and retrospective cohort studies, single-arm clinical series, registry-based analyses, and matched-pair comparisons. Studies were excluded if they were narrative reviews, systematic reviews, meta-analyses, editorials, pharmacokinetic or mechanistic overviews without patient-level oncological outcomes, or conference abstracts without an accompanying full paper. Studies focused exclusively on muscle-invasive bladder cancer, upper-tract urothelial carcinoma, or hyperthermic intraperitoneal chemotherapy for non-bladder indications were excluded. Where a study reported mixed populations, it was retained only if NMIBC outcomes were separately identifiable or constituted the dominant population. Two screening stages were applied, as depicted in Figure 1: an initial title-and-abstract screen to remove clearly irrelevant or ineligible records, followed by a full-text eligibility assessment. Both stages were performed independently and in duplicate by two reviewers (A.Cr. and V.C.-B.), who were not blinded to authorship or journal. Inter-reviewer agreement at the full-text stage was substantial (Cohen’s κ = 0.82). Disagreements were resolved by discussion and, where consensus was not reached, by adjudication by a third reviewer (A.-D.Z.); three records required adjudication. No upper limit was placed on the number of included studies, and every eligible study identified was incorporated.

### 2.4. Data Extraction and Items Collected

A structured, pre-piloted data-extraction form was applied uniformly to every included study. Data were extracted independently and in duplicate by two reviewers (A.Cr. and V.C.-B.), with discrepancies resolved by re-inspection of the source manuscript and, where necessary, adjudication by a third reviewer (A.-D.Z.). For each study, the following descriptive items were recorded: first author, year of publication, country or countries of origin, study design, single- versus multicentre status, recruitment period, and total sample size. Population descriptors captured included risk stratification (intermediate-risk, high-risk, very-high-risk, or mixed), BCG-exposure status (BCG-naive, BCG-intolerant, BCG-unresponsive, or BCG-failure), the proportion with carcinoma in situ, tumour stage and grade distribution, and median patient age where reported. Intervention details extracted comprised the heating platform or device, the chemotherapeutic agent and dose, target temperature, and the instillation schedule (number of induction and maintenance instillations). The principal outcome items collected were recurrence-free survival at defined time points (commonly 12 and 24 months), progression-free survival, complete response rate, cancer-specific survival, overall survival, bladder-preservation rate, and median follow-up duration. Safety items included the overall adverse-event rate, the proportion of severe (grade ≥ 3) events, and the rate of treatment discontinuation due to toxicity. For comparative studies, the corresponding outcomes in the comparator arm (most often BCG) and the reported statistical significance of between-group differences were also recorded. When a specific variable was not reported, could not be retrieved, or was not applicable to a given study, it was recorded as “NR” (not reported) rather than imputed or estimated. Every numerical value entered into the evidence tables was traced back to, and attributed to, the specific source study and its corresponding bibliographic citation, so that each data point in the synthesis is verifiable against the originating manuscript. Where a study reported both intention-to-treat (ITT) and per-protocol (PP) estimates, both were extracted and the ITT estimate was used as the primary figure for synthesis. Where a study reported an enrolled cohort and a smaller analysed cohort, both denominators were recorded and are distinguished in the tables. Extracted data were organised into evidence tables addressing, respectively, study and population characteristics, oncological efficacy outcomes, comparative and safety outcomes, and risk of bias. All numerical entries were audited against the source manuscripts a second time during revision.

### 2.5. Risk-of-Bias Considerations and Data Synthesis

Because the included evidence spanned a spectrum of designs from randomised controlled trials to retrospective single-arm series, risk of bias was assessed formally using validated, design-specific instruments. Randomised controlled trials were appraised with the Cochrane RoB 2 tool [17] across its five domains (randomisation process; deviations from intended interventions; missing outcome data; measurement of the outcome; selection of the reported result), with an overall judgement of low risk, some concerns, or high risk. Non-randomised comparative studies, including matched-pair and comparative retrospective cohorts, were appraised with ROBINS-I [18] across its seven domains, yielding an overall judgement of low, moderate, serious, or critical risk. Single-arm series and registry cohorts, for which ROBINS-I comparison-based domains are not applicable, were appraised with the Newcastle–Ottawa Scale [19] adapted for non-comparative designs (selection, ascertainment of exposure and outcome, adequacy and duration of follow-up), with 7–9 stars regarded as low, 5–6 as moderate, and 0–4 as high risk of bias. Two reviewers (A.Cr. and V.C.-B.) performed all appraisals independently, and disagreements were resolved by consensus with a third reviewer (A.-D.Z.). Domain-level and overall judgements are reported in Section 3.5, and the resulting judgements are carried explicitly into the interpretation of every major outcome in Section 3 and Section 4: outcomes derived exclusively from studies at serious or high risk of bias are described as hypothesis-generating rather than as evidence of comparative effectiveness. Heterogeneity across studies in heating platform, chemotherapeutic agent, dose, target temperature, instillation schedule, risk composition, and BCG-exposure status was substantial. Given this clinical and methodological diversity, a quantitative meta-analytic pooling of effect estimates was judged inappropriate, as it could generate spuriously precise summary statistics that obscure genuine between-study differences. A narrative, structured synthesis was therefore adopted. Because the eligible population spans clinically distinct groups with different prognoses, treatment objectives, and appropriate comparators, outcomes were grouped into pre-specified strata before synthesis: (i) BCG-naive or predominantly BCG-naive intermediate- and high-risk papillary disease; (ii) BCG-intolerant, BCG-failure, or BCG-unresponsive papillary disease; and (iii) carcinoma in situ, whether isolated or accompanying papillary tumours. Carcinoma in situ was analysed separately throughout and was never pooled with papillary-only disease, because response criteria (complete response) and prognosis differ fundamentally. Within each stratum, outcomes were further considered by chemotherapeutic agent, heating platform, and study design. Cross-stratum comparison of survival estimates was deliberately avoided. Where studies within the same stratum reported survival at common landmarks (12 and 24 months), these were juxtaposed descriptively to convey the range of reported outcomes; such juxtaposition is descriptive only and is not a formal indirect comparison, since follow-up duration, analytical method, risk composition, and treatment platform differ between studies. Throughout the synthesis, the strength of each inference was weighted by the design, size, and risk-of-bias judgement of the contributing studies, with randomised and large registry data accorded greater interpretive weight than small retrospective series. In line with current guidance, the absence of a statistically significant difference was not interpreted as evidence of equivalence or non-inferiority; where confidence intervals, hazard ratios, or power calculations were reported, these are presented alongside *p* values, and where they were not reported, this is stated explicitly as a limitation of the underlying study. No formal assessment of between-study consistency (I^2^ or equivalent) was possible in the absence of quantitative pooling, and no formal assessment of publication bias (funnel plot asymmetry or Egger test) was undertaken, for the same reason.

## 3. Results

### 3.1. Study Selection and Characteristics of the Included Studies

After title-and-abstract screening and full-text eligibility assessment (Figure 1), twenty-four primary clinical studies met all inclusion criteria. These comprised seven randomised controlled trials, one large multinational prospective registry, and a series of prospective and retrospective cohorts, single-arm series, and matched-pair analyses, together encompassing more than 3600 patients. Four randomised trials were added at revision, after the search was re-run and full-text accessibility was withdrawn as an eligibility criterion: a randomised comparison of radiofrequency thermochemotherapy with mitomycin C alone [20], the HIVEC-HR trial of recirculating HIVEC versus BCG [21], and the HIVEC-1 [22] and HIVEC-II [23] trials of hyperthermic versus normothermic mitomycin C. The extracted data are presented in four evidence tables: Table 1 summarises study and population characteristics; Table 2 reports oncological efficacy outcomes; Table 3 details comparative effectiveness against BCG or non-heated chemotherapy together with safety outcomes; and Table 4 reports the formal risk-of-bias assessment. Each table reports the source study and its citation for every data element, and unreported items are denoted “NR.” Because citations are numbered in order of first appearance, the methodological instruments applied in Section 2 are numbered first, the included studies are then numbered as they are introduced in Table 1 and in the text accompanying it, and every study retains its number throughout.

Table 1 shows that the twenty-four included studies, arranged chronologically from 2011 to 2026, were geographically diverse and spanned the full range of designs, from seven randomised controlled trials [20,21,22,23,26,37,43] to a multinational registry that enrolled 1028 consecutive patients, of whom 835 received mitomycin C and constituted the analysed cohort [30]; both denominators are now reported explicitly, and the enrolled and analysed figures used earlier in this review have been reconciled. Sample sizes ranged from 14 to 835 analysed patients, and the studies collectively covered every BCG-exposure category, including BCG-naive disease treated during shortage [28,41], BCG intolerance [31], and BCG-unresponsive or BCG-failure disease [34,35,38]. Mitomycin C delivered via a conductive recirculation device was the most common regimen, but epirubicin [34,41], pirarubicin [39], and gemcitabine [42] were also represented, and one study used a radiofrequency platform [27]. Most schedules followed a six-session weekly induction with monthly maintenance. The four trials added at revision are also the only studies in which HIVEC was compared with non-heated intravesical chemotherapy under randomised conditions [20,22,23] or with BCG in a population from which carcinoma in situ was excluded by protocol [21].

### 3.2. Oncological Outcomes in Papillary and Mixed-Risk Disease, by BCG-Exposure Stratum

**Figure 2:**
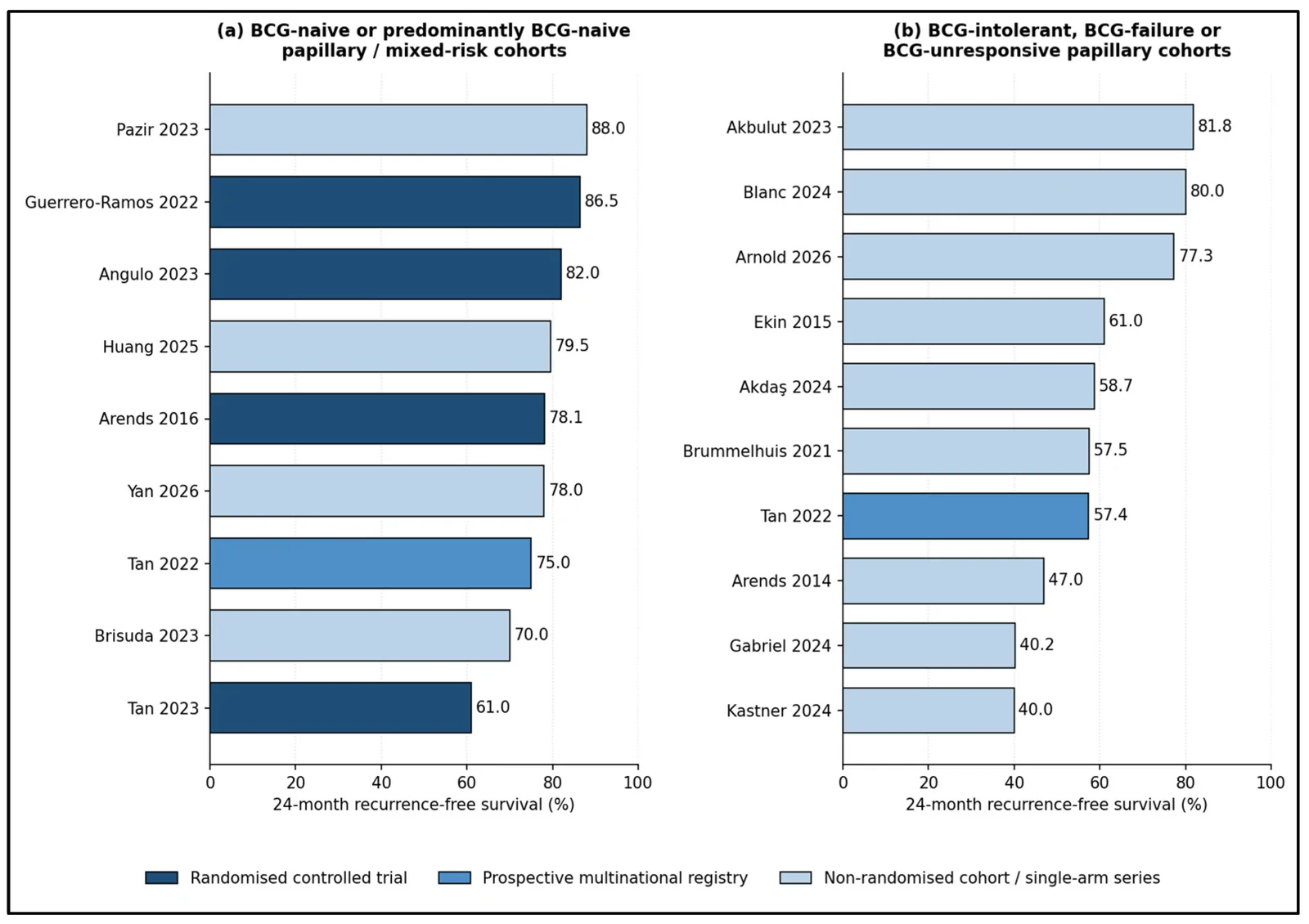
Reported 24-month recurrence-free survival in the included HIVEC studies, shown separately for (**a**) BCG-naive or predominantly BCG-naive papillary and mixed-risk cohorts, and (**b**) BCG-intolerant, BCG-failure or BCG-unresponsive papillary cohorts, with bars shaded by study design [21,22,23,24,25,26,27,30,31,32,33,34,35,36,38,39,41,42]. Values are descriptive and are not directly comparable across studies, because follow-up duration, risk composition, heating platform, and analytical method differ. No efficacy reference line is shown: the International Bladder Cancer Group consensus [9] proposes benchmark recurrence-free rates of approximately 50%, 30%, and 25% at 6, 12, and 18 months in BCG-unresponsive papillary disease and does not define a 24-month threshold.

Table 2 shows that, within the BCG-naive and mixed-risk stratum, 12-month RFS was frequently in the mid-80% to high-90% range [30,34,39], while 24-month RFS ranged from roughly 61% to 88%. Because these estimates derive from populations differing in risk composition, follow-up, heating platform, and analytical method, they are reported as a range within the stratum and were not compared statistically with one another. Progression to muscle-invasive disease was uniformly low, with several series reporting rates below 5%, including under 2% in one randomised trial [26]. The registry quantified the efficacy gradient across BCG status, with 24-month RFS falling from 75.0% in BCG-naive to 57.4% in BCG-unresponsive patients [30], and a dedicated quality-of-life cohort confirmed that the patient-reported burden of HIVEC was largely transient [40]. The distribution of reported 24-month RFS values, presented separately for the two clinically defined papillary strata and annotated by study design, is displayed in Figure 2.

As Figure 2 illustrates, reported 24-month RFS spans roughly 40% to 88% overall, but the two strata occupy overlapping rather than distinct ranges, and within each stratum the highest values come from the smallest retrospective series, while the randomised estimates sit in the middle of the distribution. The lowest values were reported by cohorts enrolling predominantly BCG-unresponsive or intensively pretreated patients. This gradient is consistent with a larger benefit in less heavily pretreated disease, but it is derived from between-study comparison of largely non-randomised cohorts and is therefore hypothesis-generating rather than confirmatory.

#### 3.2.1. BCG-Naive and Predominantly BCG-Naive Papillary Disease

Nine studies reported 24-month RFS in this stratum (Figure 2a), including five randomised trials. Arends and colleagues reported a 24-month RFS of 78.1% on intention-to-treat and 81.8% per protocol for chemohyperthermia versus 64.8% for BCG, the difference reaching statistical significance only in the per-protocol population [26]. The HIVEC-HR pilot trial, which excluded carcinoma in situ by protocol, reported a 24-month RFS of 86.5% for HIVEC versus 71.8% for BCG on intention-to-treat (*p* = 0.184) and 95.0% versus 75.1% per protocol; with 25 patients per arm and 11 recurrence events in total, the trial was designed only to inform a larger study, and the confidence interval around the effect estimate was very wide (HR 0.41, 95% CI 0.10–1.66) [21]. Progression-free survival in the same trial favoured HIVEC (95.7% versus 71.8%, *p* = 0.043) on the basis of seven events [21]. Against non-heated mitomycin C, neither HIVEC-1 (24-month RFS of 82% and 80% for 43 °C for 30 and 60 min versus 77% for normothermia; *p* = 0.6) [22] nor HIVEC-II (24-month disease-free survival of 61% versus 60%; HR 0.92, 95% CI 0.62–1.37; *p* = 0.8) [23] demonstrated any benefit from the addition of hyperthermia; both were adequately sized for their pre-specified effect and are at low risk of bias (Table 4). Non-randomised cohorts in this stratum reported 24-month RFS of 70% to 88% [32,33,39,42], and the registry reported 75.0% in its BCG-naive subgroup [30]; all of these are at moderate to serious risk of bias.

#### 3.2.2. BCG-Intolerant, BCG-Failure and BCG-Unresponsive Papillary Disease

Ten studies reported 24-month RFS in this stratum (Figure 2b), and no randomised trial is available in it. Reported values ranged from 40.0% to 81.8%. The higher figures came from small single-arm series in BCG-intolerant patients [31] or from cohorts treated with an optimised epirubicin protocol [34,41], and the lower figures from cohorts with a high proportion of BCG-unresponsive disease [35,36]. The registry, the largest source of data in this stratum, reported a 24-month RFS of 57.4% among BCG-unresponsive patients [30]. Definitions of BCG failure, relapse, intolerance and unresponsiveness were not applied uniformly across these reports, so the stratum is itself internally heterogeneous, and the consensus definitions [9] were explicitly applied in only a minority of studies. Extravesical recurrence was frequent wherever it was systematically sought, affecting 36% of patients in one cohort [34] and 23.9% in another [41]. Because every study in this stratum is non-randomised and most are single-arm, these estimates describe the outcomes achieved with HIVEC but do not establish its effect relative to any alternative, including radical cystectomy.

### 3.3. Carcinoma In Situ

**Figure 3:**
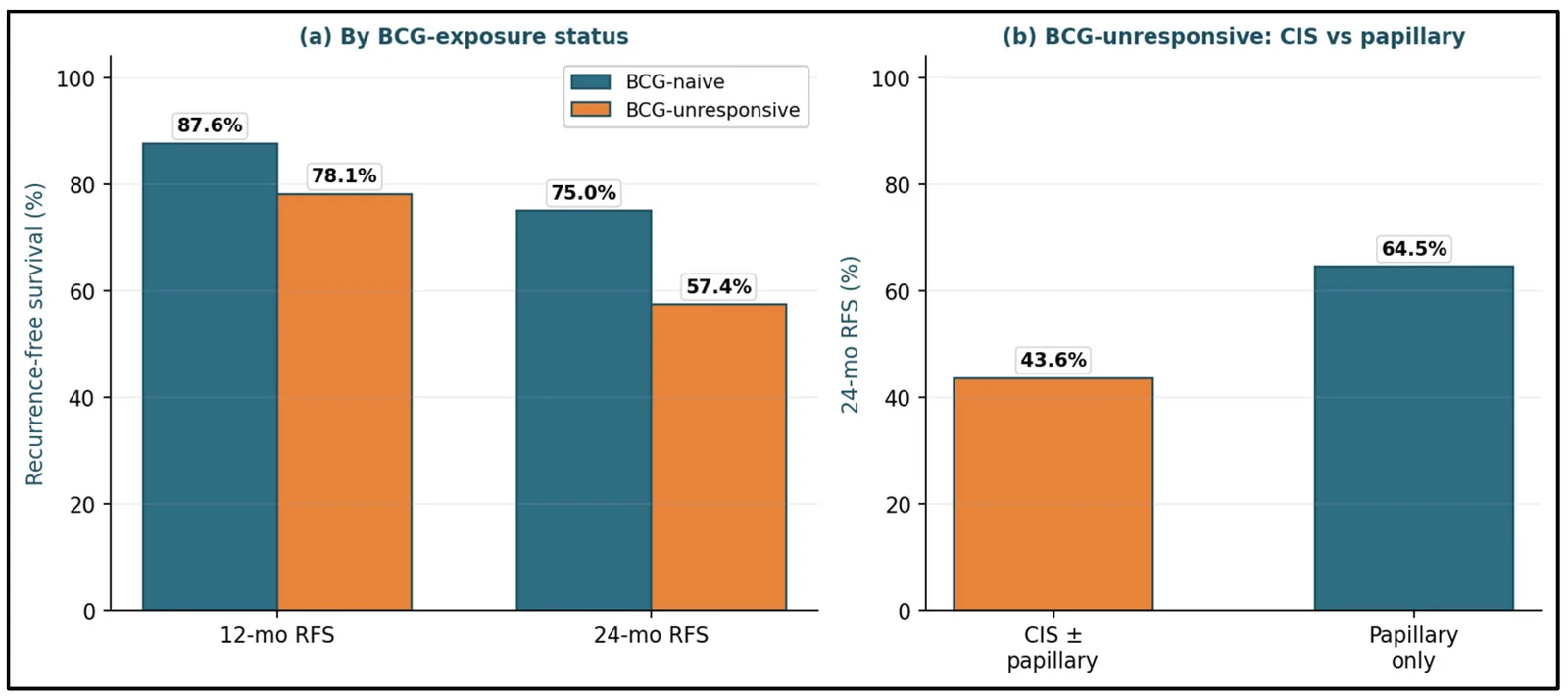
Recurrence-free survival stratified by BCG-exposure status and disease type, derived from the multinational HIVEC-E registry [30]. Panel (**a**) compares 12- and 24-month RFS in BCG-naive versus BCG-unresponsive patients; panel (**b**) compares 24-month RFS for carcinoma in situ (CIS) with/without papillary disease versus papillary-only disease.

Figure 3 isolates the registry’s stratified outcomes and conveys two patterns seen throughout the dataset. First, recurrence control was consistently higher in BCG-naive than in BCG-unresponsive disease at both 12 and 24 months. Second, within the BCG-unresponsive stratum, papillary-only disease fared substantially better than CIS-containing disease (24-month RFS of 64.5% versus 43.6%) [30]. Together the panels are consistent with preferential use of HIVEC in BCG-naive and papillary presentations, although both comparisons are internal to a single registry, are unadjusted for differences between the subgroups, and are not accompanied by confidence intervals in the source report.

Evidence specific to carcinoma in situ is sparse and is reported separately here, because response definitions (complete response rather than recurrence-free survival) and prognosis differ fundamentally from those of papillary-only disease, and the two should not be pooled. Only two included studies provide CIS-specific estimates. In the registry, patients with carcinoma in situ with or without concomitant papillary tumours had a 24-month RFS of 43.6%, compared with 64.5% for papillary-only disease within the same BCG-unresponsive stratum [30]. In the radiofrequency cohort, the 6-month complete response rate in carcinoma in situ was 56.0%, with limited long-term durability [27]. Two of the randomised trials excluded carcinoma in situ by protocol [21,22], a third enrolled intermediate-risk papillary disease only [23], and most cohort studies did not report the CIS subgroup separately. No reliable estimate of HIVEC efficacy in carcinoma in situ can therefore be derived from the present evidence base, and the outcomes reported in the papillary strata above should not be extrapolated to it.

### 3.4. Comparative Effectiveness Versus BCG and Versus Non-Heated Intravesical Chemotherapy

Table 3 shows that most comparative studies did not detect a statistically significant oncological difference between HIVEC and BCG. None was designed or powered as a non-inferiority trial, and none pre-specified a non-inferiority margin, so these findings should not be read as demonstrating non-inferiority. The randomised trial by Arends and colleagues reported superior 24-month RFS for chemohyperthermia in the per-protocol population (81.8% versus 64.8%, *p* = 0.02), whereas the corresponding intention-to-treat estimate was 78.1% [26]; the HIVEC-HR trial likewise favoured HIVEC without reaching significance on intention-to-treat (86.5% versus 71.8%, *p* = 0.184) [21]. Retrospective comparisons using gemcitabine [42], pirarubicin [39], and matched mitomycin C cohorts [32] each reported no significant survival difference versus BCG, but these studies analysed 24 to 48 HIVEC patients per arm, reported no power calculation, and are at serious risk of bias (Table 4); their null results are therefore uninformative about equivalence. Against non-heated intravesical chemotherapy, the randomised evidence is discordant: an earlier trial of radiofrequency thermochemotherapy reported a 10-year disease-free survival of 53% versus 15% for mitomycin C alone (*p* < 0.001), with a bladder preservation of 86% versus 79% [20], whereas the two contemporary conductive recirculation trials found no advantage for hyperthermia at 24 months [22,23]. Differences in heating technology, drug schedule, follow-up horizon, and era preclude any direct reconciliation of these results. In HIVEC-II, patients allocated to chemohyperthermia were also markedly less likely to complete treatment (59% versus 89%), and progression-free survival favoured the control arm on intention-to-treat (HR 3.44, 95% CI 1.09–10.82; *p* = 0.02), a signal that was no longer significant per protocol [23]. Tolerability favoured or matched HIVEC, with one trial reporting significantly fewer adverse events than BCG [42] and severe (grade ≥ 3) toxicity remaining uncommon—around 2% in the largest registry [30]. Notable cautions were a higher rate of non-healing resection-site necrosis with chemohyperthermia in one randomised trial [37] and increased toxicity without added efficacy beyond six instillations [36]. Resection completeness on second TURBT was the strongest predictor of recurrence [30], and ablative dosing improved control with radiofrequency platforms [27].

### 3.5. Risk of Bias Across the Included Studies

Table 4 shows that only two included studies were judged to be at low risk of bias overall, both of them randomised trials of hyperthermic versus normothermic mitomycin C [22,23]. Of the seven randomised trials, two were judged at low risk, and five raised some concerns, most often because of open-label delivery, small pilot sample sizes, incomplete reporting of allocation concealment, or emphasis on per-protocol rather than intention-to-treat estimates. All seven non-randomised comparative studies were judged at moderate or serious risk of bias on ROBINS-I, with confounding by indication and physician-directed treatment allocation the dominant concerns. The ten single-arm and registry cohorts scored 5 to 7 out of 9 on the Newcastle–Ottawa Scale, limited principally by the absence of a comparator, short or variable follow-up, and incomplete outcome ascertainment. These judgements are carried directly into the interpretation of each outcome reported above: the comparison with BCG rests predominantly on evidence at serious risk of bias, whereas the comparison with normothermic mitomycin C rests on the two trials at low risk of bias, neither of which found a benefit from hyperthermia.

## 4. Discussion

This systematic review of twenty-four primary clinical studies provides a consolidated picture of hyperthermic intravesical chemotherapy as a bladder-sparing strategy in NMIBC, a disease whose defining problem is recurrence despite guideline-concordant adjuvant therapy [6]. The dominant and most reproducible finding is that HIVEC achieves clinically meaningful recurrence control while keeping progression to muscle-invasive disease consistently low. The convergence of independent cohorts, randomised trials, and a large registry around this pattern is reassuring, and it accords with the favourable position assigned to device-assisted hyperthermia in recent expert syntheses of the BCG-unresponsive landscape [14,44]. That convergence is nevertheless weaker evidence than it may appear, because the contributing studies share the same design limitations, the same absence of concurrent controls, and, in several instances, the same industry sponsorship; consistency among studies at serious risk of bias does not reduce that risk. The most frequently cited comparison, a randomised trial of chemohyperthermia versus BCG, reported superior 24-month RFS only in the per-protocol population [26]; on intention-to-treat, the corresponding estimate was 78.1%, and the same pattern of a non-significant intention-to-treat result alongside a larger per-protocol effect recurred in the HIVEC-HR pilot trial [21]. Per-protocol analyses exclude patients who did not complete therapy and therefore tend to favour the arm with poorer completion; because treatment completion differed markedly between arms in HIVEC-II (59% versus 89%) [23], intention-to-treat estimates are the appropriate basis for inference in this literature and are prioritised throughout this review. Biological plausibility is supported by the demonstrated in vitro synergy between hyperthermia and intravesical cytotoxic agents [15], but plausibility is not evidence of clinical benefit, and the two adequately sized randomised comparisons with normothermic mitomycin C did not demonstrate one [22,23].

The clinical positioning of HIVEC is best understood across the spectrum of BCG-exposure status, which depends on the standardised definitions articulated by the International Bladder Cancer Group [9]. In BCG-naive intermediate- and high-risk papillary disease, the available randomised and observational data are compatible with recurrence control of a similar order to induction BCG, which makes HIVEC a reasonable substitute when BCG is unavailable—particularly because the activity and toxicity of BCG itself remain difficult to standardise [8]. The evidence does not, however, establish equivalence: no trial has been powered to exclude a clinically important difference, and the confidence intervals reported in the two randomised comparisons with BCG are wide enough to remain compatible with a meaningful advantage for either treatment [21,26]. In BCG-intolerant patients, focused cohorts show effective bladder-sparing control with a gentler toxicity profile [31]. The most difficult setting is BCG-unresponsive disease, where the guideline standard is radical cystectomy [6]; here HIVEC delivers more modest but still valuable control, with a clear gradient favouring papillary-only over CIS disease. Outcomes in this setting are often set alongside those of newer approved alternatives—pembrolizumab [10], nadofaragene firadenovec [11], sequential gemcitabine–docetaxel [12], and the drug-releasing system TAR-200 [45]. Any such comparison is indirect and should be read with caution: those agents were evaluated in single-arm trials using centrally reviewed, protocol-defined complete-response and disease-free endpoints in strictly defined BCG-unresponsive populations, whereas the HIVEC cohorts used heterogeneous eligibility definitions, local outcome assessment, and mixed BCG-exposure categories. No head-to-head trial and no matched or adjusted indirect comparison exists, so the juxtaposition provides descriptive context rather than evidence of comparable efficacy.

Tolerability and health-economic considerations further strengthen the case for HIVEC. Severe adverse events were uncommon across the dataset, and patient-reported data indicate that the quality-of-life impact is largely transient [40]. This matters not only for patient experience but for treatment completion, since BCG schedules are frequently truncated by intolerance in routine practice, as documented in contemporary quality-indicator registries [13]. The contrast with radical cystectomy is salient: cystectomy, though oncologically definitive, carries substantial perioperative morbidity and long-term functional consequences that many older or comorbid patients cannot accept [2], which is precisely why bladder-sparing options command such interest. The review also surfaced an actionable determinant of success: the completeness of the initial resection, indexed by tumour on restaging TURBT, was the single strongest predictor of recurrence and progression in the largest dataset [30], so meticulous, fully staged resection—aided where available by enhanced cystoscopy [4]—is as important as the choice of HIVEC itself.

A question distinct from the comparison with BCG is whether heating adds anything to intravesical chemotherapy itself, and the randomised evidence on this point is now the most methodologically secure part of the literature—and the least encouraging. The original randomised comparison of radiofrequency thermochemotherapy with mitomycin C alone reported a large and durable advantage, with a 10-year disease-free survival of 53% versus 15% (*p* < 0.001) and bladder-preservation rates of 86% versus 79% [20]. Two subsequent trials using conductive recirculation did not reproduce this advantage: HIVEC-1 found 24-month recurrence-free survival of 82% and 80% with 30 and 60 min of hyperthermia versus 77% with normothermic mitomycin C (*p* = 0.6) [22], and HIVEC-II found 24-month disease-free survival of 61% versus 60% (HR 0.92, 95% CI 0.62–1.37; *p* = 0.8), with progression-free survival numerically favouring the control arm [23]. Several explanations are plausible and mutually compatible: the platforms differ in whether the bladder wall is heated directly or only by conduction from the instillate; the historical trial used a longer and more intensive schedule; and the contemporary trials enrolled intermediate-risk patients in whom the absolute recurrence risk, and therefore the scope for benefit, is lower. Whatever the explanation, the practical implication is that the incremental value of hyperthermia over conventional intravesical chemotherapy cannot currently be regarded as established for conductive recirculation devices.

The included studies used several distinct heating technologies that are frequently discussed interchangeably but are not equivalent, and the differences between them bear directly on how the evidence should be pooled and interpreted. Table 5 compares the platforms represented in this review. The principal distinction is between intravesical radiofrequency systems, which heat the bladder wall directly with a microwave applicator and measure wall temperature with mounted thermocouples, and conductive recirculation systems, which heat the drug solution externally and rely on conduction from the instillate, so that the bladder-wall temperature is inferred rather than measured. The two randomised trials that showed an advantage over comparator therapy used a radiofrequency platform [20,26], whereas the two that showed none used a conductive system [22,23]—an association that is confounded by era, schedule, and risk group but that cannot be dismissed. Radiofrequency platforms carry the largest and longest evidence base and provide direct thermometry, at the cost of a substantially more expensive console and a dedicated catheter; conductive systems are portable, simpler to operate, and considerably cheaper, which explains their rapid adoption during the BCG shortage [30]. Cross-platform pooling of outcomes should therefore be avoided, and future trials should report the heating technology, the measured or estimated bladder-wall temperature, and the achieved thermal dose rather than the nominal device setting alone [46,47,48].

Any appraisal of HIVEC must also account for what its delivery requires, since these demands shape feasibility far more than the marginal differences in reported recurrence rates. Three resource commitments are unavoidable. First, a heating console must be purchased or leased; published estimates place radiofrequency consoles at roughly €90,000 and conductive systems at roughly €42,000, although current prices vary by market and procurement model [47]. Second, every session consumes a dedicated single-use catheter, reported at approximately €600 for radiofrequency triple-lumen catheters and approximately €200 for conductive three-way catheters, so consumable cost scales directly with the number of instillations and with any decision to extend maintenance [47]. Third, nursing and medical personnel require specific training in device operation, catheterisation, temperature monitoring, and cytotoxic-spill management, and competence must be maintained in centres treating small numbers of patients. Beyond cost, the treatment is time-consuming and uncomfortable: a single instillation occupies 30 to 60 min of recirculation, in addition to preparation and recovery, and requires a dedicated room and supervised chair time for each of six induction and up to six maintenance sessions. Local symptoms are common and directly attributable to the procedure, with bladder spasms reported in 29.2% of HIVEC patients in HIVEC-HR [21], haematuria in 21% of patients receiving more than six instillations [36], and adverse events rising from 33% to 48% as dwell time increased from 30 to 60 min in HIVEC-1 [22]. These burdens have measurable consequences for delivery: only 59% of patients randomised to chemohyperthermia in HIVEC-II completed treatment, against 89% of controls [23]; approximately 5% discontinued in the registry [30]; and 13 of 67 patients withdrew in a dedicated quality-of-life cohort [40]. Centres considering HIVEC should therefore weigh capital outlay, per-session consumable cost, training requirements, throughput, and patient tolerance alongside the oncological data, and should counsel patients explicitly about session length and expected discomfort.

Several tensions temper enthusiasm and define the research agenda. The agent, heating platform, target temperature, and instillation schedule varied widely, and the data do not establish the optimal combination; one multicentre retrospective analysis found no advantage beyond six instillations, at the cost of more toxicity [36], while another flagged non-healing resection-site necrosis with chemohyperthermia [37]. The predominance of retrospective, single-arm designs introduces selection bias and confounding by indication. The cautionary experience of trials with control arms—where thermochemotherapy after BCG recurrence did not always outperform further intravesical therapy—reminds us that single-arm efficacy signals can overstate comparative benefit [49]. The recurrent observation of extravesical recurrence among BCG-failure patients treated with HIVEC highlights that bladder-focused therapy does not address the whole urothelial field and mandates whole-tract surveillance [41]. Looking forward, integrating molecular biomarkers to identify responders [5] and head-to-head trials against both BCG and the agents reshaping urothelial cancer care more broadly [50] will be essential to convert a promising alternative into a firmly evidence-based standard.

## 5. Study Limitations

This review has several limitations. First, the included evidence remained dominated by retrospective and single-arm designs; although seven randomised controlled trials are now included, four of them enrolled fewer than 110 patients in total, and only two were judged at low risk of bias, so the synthesis is vulnerable to selection bias, confounding by indication, and variable follow-up. Second, substantial clinical and methodological heterogeneity—in chemotherapeutic agent, heating platform, dose, target temperature, instillation schedule, risk composition, and BCG-exposure status—precluded a quantitative meta-analysis, so outcomes were synthesised narratively, and the reported survival figures should be read as a range rather than a pooled estimate. Third, definitions of key constructs, particularly BCG-unresponsive disease and the boundaries between BCG intolerance, relapse, and failure, were not uniform across all studies despite the availability of consensus definitions, limiting cross-study comparison in the very subgroups where benchmarking is most consequential. Fourth, although full-text accessibility was withdrawn as an eligibility criterion at revision and no study was ultimately excluded on that ground, the search remained confined to PubMed and PubMed Central and did not interrogate Embase, Scopus, Web of Science, the Cochrane Library, or trial registries, so eligible reports indexed only elsewhere, published in languages poorly covered by PubMed, or reported solely as abstracts may still have been missed. Fifth, several included studies had modest sample sizes, short or incomplete follow-up, and missing data for specific landmark outcomes (denoted NR), which constrains the strength of certain inferences. Sixth, the review was not prospectively registered in PROSPERO and no protocol was published in advance, so protocol deviations cannot be independently verified. Seventh, no quantitative synthesis was undertaken; consequently, no formal assessment of between-study statistical consistency (I^2^, τ^2^, or prediction intervals) and no formal assessment of small-study effects (funnel-plot asymmetry or Egger test) could be performed, and the ranges reported here must not be read as pooled estimates. Eighth, several comparative studies reported *p* values without confidence intervals, effect estimates, or power calculations; a null result in such studies is uninformative about equivalence, and the frequent absence of a statistically significant difference between HIVEC and BCG in this literature should not be mistaken for evidence that the treatments are equivalent. Finally, publication bias toward positive findings cannot be excluded. These limitations collectively argue for cautious interpretation and for prioritising adequately powered prospective randomised trials with standardised protocols.

## 6. Conclusions

Hyperthermic intravesical chemotherapy is a generally well-tolerated, bladder-sparing option for selected patients with non-muscle-invasive bladder cancer, particularly during BCG shortage or when BCG is unsuitable. In BCG-naive intermediate- and high-risk papillary disease, recurrence control of a similar order to BCG has been reported, but the evidence base is dominated by non-randomised studies at moderate to serious risk of bias, the randomised signal favouring HIVEC over BCG derives principally from per-protocol analyses, and two adequately sized randomised trials found no benefit from adding hyperthermia to normothermic mitomycin C. Equivalence or non-inferiority to BCG therefore cannot be claimed on the present evidence. After BCG failure, disease control is clinically meaningful but less durable, and no reliable estimate is available for carcinoma in situ, which should not be managed on the basis of data derived from papillary disease. Implementation additionally requires capital investment in a heating console, a single-use catheter for every session, trained personnel, and prolonged, frequently uncomfortable treatment sessions with imperfect completion rates. Adequately powered randomised trials with standardised protocols, pre-specified non-inferiority margins, stratification by BCG-exposure status and by the presence of carcinoma in situ, and intention-to-treat reporting are needed before HIVEC can be positioned as an established alternative to BCG.

## Figures and Tables

**Table 1:** Characteristics of included studies and patient populations.

Study [Ref.]	Year	Country/Design	*N*	Population/Risk and BCG Status	Agent/Device and Schedule
Colombo et al. [20]	2011	Italy/Israel; RCT	83 (42 CHT)	Intermediate/high-risk papillary; adjuvant after complete TURBT	RF-CHT (Synergo) MMC vs. MMC alone
Arends et al. [24]	2014	Netherlands; prospective single-centre	160	NMIBC; 81% prior BCG	MMC/epirubicin chemohyperthermia
Ekin et al. [25]	2015	Turkey; two-centre retrospective	43	High-risk; BCG contraindicated/intolerant	MMC chemohyperthermia
Arends et al. [26]	2016	Multinational; RCT	95 (CHT)	Intermediate/high-risk papillary	CHT-MMC vs. BCG
Brummelhuis et al. [27]	2021	Netherlands; cohort	274 (efficacy)	Intensively pretreated; CIS and papillary	RF-CHT (Synergo); MMC/epirubicin
Grimberg et al. [28]	2021	USA; single-arm expanded access	14	Intermediate/high-risk; BCG shortage	High-dose MMC (120 mg) HIVEC, 43 °C
Kostyev et al. [29]	2021	Ukraine; comparative retro-prospective	53 (HIVEC)	High-risk NMIBC	HIVEC vs. BCG (54)
Tan et al. [30]	2022	Multinational (HIVEC-E); registry	835	BCG-naive 66.7%, unresp. 20.6%, failure 8.9%	MMC, Combat BRS, 43 °C
Guerrero-Ramos et al. [21]	2022	Spain; RCT (HIVEC-HR)	25 (HIVEC)	High-risk papillary; CIS excluded; predominantly BCG-naive	MMC 40 mg, Combat BRS, 43 °C vs. BCG (25)
Akbulut et al. [31]	2023	Turkey; retrospective	22	High-risk papillary; BCG-intolerant	HIVEC-MMC
Pazir et al. [32]	2023	Turkey; matched-pair retrospective	24 (HIVEC)	High-risk papillary	HIVEC-MMC vs. BCG (24), matched
Brisuda et al. [33]	2023	Czech Republic; single-arm	47	High- and very-high-risk; BCG unavailable	HIVEC ± maintenance
Angulo et al. [22]	2023	Spain; RCT (HIVEC-1)	213 (HIVEC)	Intermediate-risk papillary	MMC 40 mg, Combat BRS, 43 °C for 30 vs. 60 min vs. normothermic MMC (106)
Tan et al. [23]	2023	UK; RCT (HIVEC-II)	131 (CHT)	Intermediate-risk papillary	MMC 40 mg, Combat BRS, 43 °C vs. normothermic MMC (128)
Blanc et al. [34]	2024	Switzerland; dual-centre retrospective	39	Recurrence after failed BCG; high-risk	Epirubicin HIVEC (optimised)
Kastner et al. [35]	2024	Germany; single-centre retrospective	60	68.6% post-BCG; 55% BCG-unresponsive	HIVEC; 6 induction + 6 maintenance
Gabriel et al. [36]	2024	France; multicentre retrospective	261	NMIBC incl. BCG-unresponsive	HIVEC; 6 vs. >6 instillations
Sachan et al. [37]	2024	India; RCT (3-arm)	45 (C-HT)	Low-grade intermediate-risk	C-HT MMC vs. MMC vs. BCG
Akdaş et al. [38]	2024	Turkey; retrospective	59	T1 high-grade; BCG relapse/intolerant	CHT-MMC; 6 + 6
Huang et al. [39]	2025	China; retrospective comparative	48 (HIVEC)	High-risk NMIBC	Pirarubicin HIVEC vs. BCG (43)
Iossa et al. [40]	2025	Italy; prospective QoL cohort	67	High-risk; BCG failure/intolerance	HIVEC; QoL endpoints
Arnold et al. [41]	2026	Switzerland; multicentre retrospective	88	BCG-naive 26%, BCG-failure 74%	Epirubicin HIVEC
Yan et al. [42]	2026	China; single-centre retrospective	38 (HIVEC)	Intermediate/high-risk	Gemcitabine HIVEC vs. BCG (47)
Michael et al. [43]	2026	India; RCT (3-arm)	25 (HIVEC)	Intermediate/high-risk	Gem/HIVEC-MMC/BCG

BCG: Bacillus Calmette–Guérin; CHT: chemohyperthermia; HIVEC: hyperthermic intravesical chemotherapy; MMC: mitomycin C; NMIBC: non-muscle-invasive bladder cancer; RCT: randomised controlled trial; RF-CHT: radiofrequency-induced chemohyperthermia.

**Table 2:** Oncological efficacy outcomes of HIVEC across included studies.

Study [Ref.]	Median FU (mo)	12 mo RFS	24 mo RFS	PFS	Other Efficacy/Response
Colombo et al. [20]	91	NR	NR	NR	10 yr DFS 53% vs. 15% for MMC alone (*p* < 0.001); bladder preservation 86% vs. 79%
Arends et al. [24]	75.6	60%	47%	Progression 4%	Highly recurrent disease lower RFS (HR 2.40)
Ekin et al. [25]	30	82%	61%	NR	Median RFS 23 mo; recurrence 32.5%
Arends et al. [26]	NR	NR	78.1% ITT/81.8% PP	Progression < 2%	vs. BCG 64.8% 24 mo RFS (PP *p* = 0.02)
Brummelhuis et al. [27]	NR	77.9% (papillary)	57.5%	NR	CIS 6 mo CR 56.0%; 5 yr papillary RFS 37.2%
Grimberg et al. [28]	11	NR	NR	NR	2/14 recurrence after early discontinuation
Kostyev et al. [29]	NR	NR	NR	NR	Recurrence 17% vs. 39% BCG (*p* = 0.012)
Tan et al. [30]	22.4	85.0%	70.0%	2 yr 90.8%	BCG-naive 24 mo RFS 75.0%; unresp. 57.4%; CR 90.4%
Guerrero-Ramos et al. [21]	33.7	NR	86.5% ITT/95.0% PP	2 yr 95.7% ITT	BCG arm 71.8% ITT (*p* = 0.184); HR 0.41 (95% CI 0.10–1.66); CSS 100%
Akbulut et al. [31]	32.2	NR	81.8% (overall)	95.4%	Mean time to recurrence 29.2 mo
Pazir et al. [32]	28	NR	88%	2 yr 94%	Matched vs. BCG (83% RFS, 100% PFS); NS
Brisuda et al. [33]	32	84%	70%	2 yr 89%	48 mo RFS 59%
Angulo et al. [22]	24	NR	82% (43 °C/30 min)/80% (60 min) ITT	6 progressions (ITT)	Normothermic MMC 77% ITT (*p* = 0.6); PP 83%/80% vs. 77% (*p* = 0.59)
Tan et al. [23]	24	NR	61% DFS ITT	HR 3.44 favouring control (*p* = 0.02)	Control 60% DFS; HR 0.92 (95% CI 0.62–1.37; *p* = 0.8); completion 59% vs. 89%
Blanc et al. [34]	28	94.8%	80%	NR	Extravesical recurrence 36%; cystectomy 18%
Kastner et al. [35]	12	67%	40%	2 yr 98%	Bladder preservation ~80%
Gabriel et al. [36]	25.5	NR	40.2%	2 yr 86%	No RFS/PFS difference 6 vs. >6 instillations
Sachan et al. [37]	26	NR	NR	NR	Time to recurrence 13.6 mo; NS vs. MMC/BCG
Akdaş et al. [38]	44	NR	58.7%	2 yr 72.6%	44 mo RFS 48%; PFS 66.2%
Huang et al. [39]	61.0	98.0%	79.5%	2 yr 89.6%	5 yr RFS 50.0%; 5 yr OS 90.4%
Iossa et al. [40]	9	NR	NR	NR	QoL: transient symptom increase; activity preserved
Arnold et al. [41]	38	84.1%	77.3%	2 yr 93.2%	Progression 9.1%; extravesical recurrence 23.9%
Yan et al. [42]	NR	NR	78.0%	2 yr 91.7%	CSS not different vs. BCG (75.0% RFS)
Michael et al. [43]	12	84%	NR	No progression	Gem 94%, BCG 92% 12 mo RFS; *p* = 0.675

CR: complete response; CSS: cancer-specific survival; FU: follow-up; NR: not reported; PFS: progression-free survival; RFS: recurrence-free survival.

**Table 3:** Comparative effectiveness versus BCG and safety/tolerability outcomes.

Study [Ref.]	Comparator and Key Result	Any AE Rate	Grade ≥ 3	Tolerability Notes
Colombo et al. [20]	MMC alone (RCT); 10 yr DFS 53% vs. 15% (*p* < 0.001)	NR	NR	Synergo RF-CHT; bladder preservation 86% vs. 79%
Arends et al. [24]	Epirubicin vs. MMC (2 yr RFS 55% vs. 46%, *p* = 0.30)	NR	NR	Earlier CHT associated with higher RFS
Ekin et al. [25]	Single-arm	53%	NR	Mostly grade 1–2
Arends et al. [26]	BCG RCT; 24 mo RFS 81.8% vs. 64.8% (*p* = 0.02)	NR	NR	No new safety concerns
Brummelhuis et al. [27]	Adjuvant vs. ablative dose	NR	NR	Ablative dose lower recurrence (HR 0.54)
Grimberg et al. [28]	Single-arm high-dose	50%	0%	79% tolerated; MMC allergy 29%
Kostyev et al. [29]	BCG; recurrence 17% vs. 39% (*p* = 0.012)	NR	NR	Lower Ki-67 in HIVEC recurrences
Tan et al. [30]	Single-arm registry	25.6% minor	2.0%	~5% discontinued; 2nd-TURBT tumour key predictor
Guerrero-Ramos et al. [21]	BCG (RCT); 24 mo RFS 86.5% vs. 71.8% ITT (*p* = 0.184)	64.6% any AE	16.7% vs. 16.7%	Grade 4–5 events only in BCG arm; bladder spasms 29.2% with HIVEC
Akbulut et al. [31]	Single-arm	50%	NR	95% of AEs mild-to-moderate
Pazir et al. [32]	BCG matched-pair; RFS/PFS NS	50.0% vs. 54% BCG	NR	Safety similar to BCG (*p* = 0.77)
Brisuda et al. [33]	Single-arm	19.1% transient LUTS	NR	1 salvage cystectomy
Angulo et al. [22]	Normothermic MMC (RCT); 24 mo RFS 82%/80% vs. 77% (*p* = 0.6)	33% vs. 35% vs. 48% (*p* = 0.05)	SAE 8.1%	More adverse events with the 60-min dwell; no QoL deterioration
Tan et al. [23]	Normothermic MMC (RCT); 24 mo DFS 61% vs. 60% (*p* = 0.8)	87 vs. 77 patients	Mostly low grade	Completion 59% vs. 89%; ITT PFS favoured control (HR 3.44, *p* = 0.02)
Blanc et al. [34]	Single-arm	No AE > grade 2	0%	No events above grade 2
Kastner et al. [35]	BCG context	NR	NR	Better tolerability vs. BCG
Gabriel et al. [36]	6 vs. >6 instillations	Haematuria 21% vs. 8.5%	3.1%	More toxicity with >6 cycles
Sachan et al. [37]	MMC and BCG; recurrence NS	NR	NR	Non-healing necrosis 22.2% with C-HT
Akdaş et al. [38]	Single-arm	NR	NR	Option for BCG-unresponsive/intolerant
Huang et al. [39]	BCG; RFS/PFS/OS NS (*p* > 0.4)	Comparable to BCG	NR	Similar severity to BCG
Iossa et al. [40]	Single-arm QoL	13/67 discontinued	NR	Transient QoL impact
Arnold et al. [41]	Single-arm; BCG-naive and BCG-failure strata	NR	NR	Extravesical recurrence 23.9%; whole-tract surveillance advised
Yan et al. [42]	BCG; RFS/PFS/CSS NS	47.4% vs. 70.2% BCG	NR	Fewer AEs with HIVEC (*p* = 0.033)
Michael et al. [43]	BCG and gemcitabine; 12 mo RFS NS	NR	NR	BCG most toxic; gemcitabine best tolerated

AE: adverse event; BCG: Bacillus Calmette–Guérin; CHT: chemohyperthermia; HIVEC: hyperthermic intravesical chemotherapy; MMC: mitomycin C; NR: not reported.

**Table 4:** Formal risk-of-bias assessment of the included studies.

Study [Ref.]	Design	Tool	Overall Judgement	Principal Domains of Concern
Colombo et al. [20]	RCT	RoB 2	Some concerns	Open label; long-term re-analysis of 65/75 original patients; allocation concealment not described
Arends et al. [26]	RCT	RoB 2	Some concerns	Open label; premature closure and under-accrual; emphasis on per-protocol result
Guerrero-Ramos et al. [21]	RCT (pilot)	RoB 2	Some concerns	n = 50; open label; imbalance in prior intravesical therapy; very wide confidence intervals
Angulo et al. [22]	RCT	RoB 2	Low	Open label, but adequate randomisation, pre-specified primary outcome, ITT and PP both reported
Tan et al. [23]	RCT	RoB 2	Low	Registered protocol, ITT primary analysis; differential treatment completion between arms
Sachan et al. [37]	RCT	RoB 2	Some concerns	Small three-arm trial; randomisation and concealment not fully described; no power calculation
Michael et al. [43]	RCT	RoB 2	Some concerns	Small arms (n = 25); short follow-up; limited reporting of randomisation
Brummelhuis et al. [27]	Comparative cohort	ROBINS-I	Serious	Long recruitment window; adjuvant versus ablative dose not randomised; mixed pretreatment
Kostyev et al. [29]	Comparative cohort	ROBINS-I	Serious	Retro-prospective allocation; confounding by indication; no multivariable adjustment
Pazir et al. [32]	Matched-pair	ROBINS-I	Moderate	Matching mitigates some confounding; residual confounding and small sample
Gabriel et al. [36]	Comparative cohort	ROBINS-I	Serious	Number of instillations determined by tolerability; immortal-time and indication bias
Huang et al. [39]	Comparative cohort	ROBINS-I	Serious	Retrospective allocation; no adjustment for baseline imbalance
Arnold et al. [41]	Comparative cohort	ROBINS-I	Serious	Retrospective multicentre; heterogeneous BCG-exposure strata; no concurrent comparator
Yan et al. [42]	Comparative cohort	ROBINS-I	Serious	Single-centre retrospective; clinician-directed treatment selection
Arends et al. [24]	Single-arm	NOS	6/9 (moderate)	Single-centre retrospective series; heterogeneous prior therapy
Ekin et al. [25]	Single-arm	NOS	5/9 (moderate)	Small two-centre retrospective series; incomplete outcome reporting
Grimberg et al. [28]	Single-arm	NOS	6/9 (moderate)	n = 14; expanded access; early discontinuation; 11-month follow-up
Tan et al. [30]	Registry	NOS	7/9 (low-moderate)	Prospective registry; 835 of 1028 enrolled analysed; variable follow-up; industry support
Akbulut et al. [31]	Single-arm	NOS	5/9 (moderate)	n = 22; retrospective; no comparator
Brisuda et al. [33]	Single-arm	NOS	6/9 (moderate)	Single-arm; no comparator; selected population treated during BCG unavailability
Blanc et al. [34]	Single-arm	NOS	6/9 (moderate)	Dual-centre retrospective; optimised protocol applied selectively
Kastner et al. [35]	Single-arm	NOS	5/9 (moderate)	Short median follow-up (12 months); mixed BCG-exposure status
Akdaş et al. [38]	Single-arm	NOS	5/9 (moderate)	Small retrospective series; no comparator
Iossa et al. [40]	Single-arm (QoL)	NOS	6/9 (moderate)	Quality-of-life endpoints; 9-month follow-up; 13 of 67 discontinued

NOS: Newcastle–Ottawa Scale; RoB 2: Cochrane risk-of-bias tool for randomised trials, version 2; ROBINS-I: Risk Of Bias In Non-randomised Studies of Interventions. Overall judgements: RoB 2—low risk, some concerns, or high risk; ROBINS-I—low, moderate, serious, or critical; NOS—7–9 stars low, 5–6 moderate, 0–4 high risk of bias.

**Table 5:** Comparison of the bladder-hyperthermia delivery platforms represented in the included studies.

Platform (Manufacturer)	Heating Mechanism	Catheter/Consumable	Target Temperature	Evidence Base and Practical Considerations
Synergo SB-TS 101/RITE (Medical Enterprises, Amsterdam, The Netherlands)	Intravesical 915 MHz microwave/radiofrequency applicator heating the bladder wall directly	Dedicated single-use 20F triple-lumen catheter with integrated antenna and thermocouples	42 ± 2 °C at the bladder wall	Largest and longest evidence base, including randomised comparisons with MMC alone [20], with BCG [26] and after BCG failure [49]; direct thermometry; highest console and catheter cost
Combat BRS (Combat Medical, Wheathampstead, UK)	External aluminium heat exchanger with closed-loop recirculation of the heated drug solution (conductive)	Single-use 16F three-way Foley catheter with temperature sensor	43 ± 0.5 °C in the recirculating fluid	Used in HIVEC-1 [22], HIVEC-II [23], HIVEC-HR [21] and the HIVEC-E registry [30]; portable and lowest capital cost; bladder-wall temperature inferred, not measured
Unithermia (Elmedical, Hod-Hasharon, Israel)	External conical heat exchanger with conductive recirculation	Single-use 18F three-way probe with temperature sensor	Up to 46.5 °C in the console; approximately 44.5 °C delivered	Lower console and catheter cost than radiofrequency systems; limited long-term outcome data and no randomised evidence in NMIBC
BR-TRG-I/BR-PRG (Bright Medical Technology, Guangzhou, China)	Water-bath heating of the drug with dual-filtration recirculation (conductive)	Single-use 20F three-way Foley catheter with temperature sensor	Approximately 45 °C in the water bath	Basis of the pirarubicin and gemcitabine series reported from China [39,42]; evidence largely single-centre and retrospective
Loco-regional external applicators (BSD-2000, Pyrexar Medical, Salt Lake City, UT, USA; AMC 70 MHz)	External electromagnetic deep-regional heating of the pelvis	Standard catheter; no dedicated single-use consumable	41–43 °C regionally	Greater depth of penetration but heats surrounding pelvic tissue; not represented among the NMIBC studies included in this review

BCG: Bacillus Calmette–Guérin; MMC: mitomycin C; NMIBC: non-muscle-invasive bladder cancer; RITE: radiofrequency-induced thermochemotherapy effect. Device specifications are taken from published technical descriptions and comparative overviews [46,47,48].

## Data Availability

The data supporting the findings of this systematic review are available from the cited primary studies.

## Supplementary Materials

The following supporting information can be downloaded at: https://www.mdpi.com/article/10.3390/jcm15166352/s1, Table S1: PRISMA 2020 checklist.

## Abbreviations

The following abbreviations are used in this manuscript: AE, adverse event; BCG, Bacillus Calmette–Guérin; CIS, carcinoma in situ; CHT, chemohyperthermia; HIVEC, hyperthermic intravesical chemotherapy; MMC, mitomycin C; NMIBC, non-muscle-invasive bladder cancer; PFS, progression-free survival; RCT, randomised controlled trial; RFS, recurrence-free survival.
